# Back to *Tityus serrulatus* Lutz & Mello, 1922 (Scorpiones: Buthidae): new comments about an old species

**DOI:** 10.1590/1678-9199-JVATITD-2022-0016

**Published:** 2022-07-13

**Authors:** Wilson R. Lourenço

**Affiliations:** 1 Muséum national d’Histoire naturelle, Sorbonne Universités, Institut de Systématique, Evolution, Biodiversité (ISYEB), UMR7205-CNRS, MNHN, UPMC, EPHE, CP 53, 57 rue Cuvier, 75005 Paris, France.

**Keywords:** Scorpion, Tityus serrulatus, Subpopulations, Mutations

## Abstract

A synopsis on the historical, geographical and ecological aspects related to the most conspicuous scorpion species of the genus *Tityus* known from Brazil is proposed. *Tityus serrulatus* Lutz & Mello, 1922 was described precisely one century ago, nevertheless many questions related to its ecological adaptations and geographical expansion remain without a precise response. This species, well known for its infamous reputation of noxious species, is also known for its capacity to reproduce asexually, by parthenogenesis. Although the individuals of a given population are considered clones, a new hypothesis could suggest the occurrence of mutations within isolated individuals, leading to distinct subpopulations that could present better phenotypic performances in ecological habitats distinct from those of the original area of distribution of the species.

## Background

**Figure 1.**
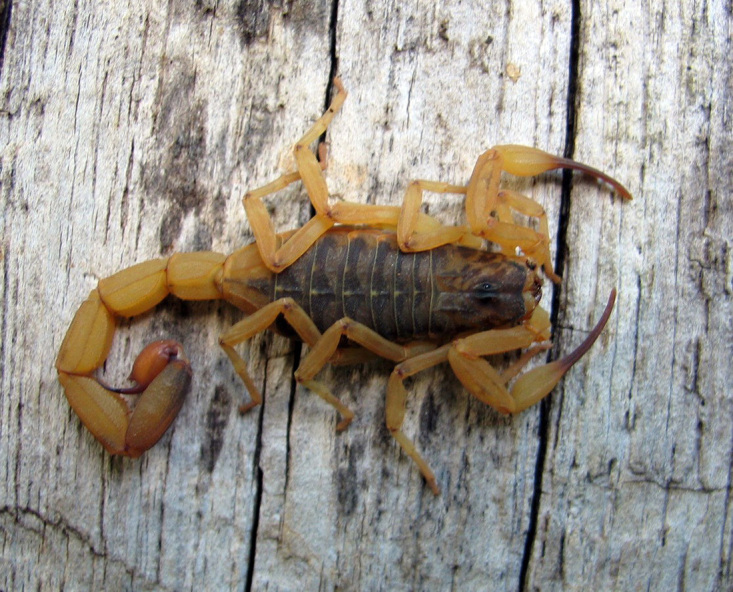
*Tityus serrulatus*. Female from Bahia state, Brazil (photo courtesy of Tiago J. Porto).

*Tityus serrulatus* a scorpion of the family Buthidae C. L. Koch, 1837, is undoubtedly the most well-known species in Brazil and South America, but equally the most infamous one (Fig. 1). The species was described precisely one century ago in 1922 by two medical researchers, Adolpho Lutz and Oswaldo de Mello Campos [1], who were little concerned with the taxonomic position of scorpions in general, but rather interested in their possible medical significance. Consequently, the original description was very succinct and devoid of any illustrations, and the original locality was indicated as Belo Horizonte in the state of Minas Gerais, in Brazil. Just a few years after the original description of *Tityus serrulatus* one of the authors, Mello Campos [2], referred to the species again in a large publication where he compiled the known information about the scorpions of Brazil. The information he gave about *T. serrulatus* was basically the same proposed by Lutz and Mello in 1922 [1], with only some additions suggesting that the species was particularly common in the city of Belo Horizonte. Subsequently, *T. serrulatus* was mainly the subject of reports referring to the incidents caused by its stings in human population; venomological aspects and serum production [3-6].

Only several decades after the description of *T. serrulatus*, the research efforts of a Brazilian zoologist, Fabio A. Matthiesen, demonstrated that this species could reproduce asexually, by parthenogenesis, what partially explained the great capacity of the species to colonize new modified environments [7]. Nevertheless, the huge capacity of this species for expanding geographically continued to be regarded as a casual factor simply associated with human migrations and its ability to adapt to anthropic environments. Only many years later, Lourenço [8] proposed, for the first time, to associate problems of scorpionism with aspects of the life history strategies in different species. Subsequently, this question was further developed in association with parthenogenesis [9-14].

Since the preliminary publication on biogeography, life history strategies and scorpionism [8] many new objective efforts have been made trying to explain possible patterns of distribution, adaptation and even evolution of *T. serrulatus* [13]. Nevertheless, in parallel, many imprecise publications related to taxonomical aspects of *T. serrulatus* and associated species, or bringing imprecise ranges of distribution for these same species, were published [15-17].

Some other publications proved to be even more peculiar by suggesting “sensationalistic titles” such as “Selected to survive and kill: *Tityus serrulatus* the Brazilian yellow scorpion” [18]; however, much of the offered information is based on already known data from previous publications and in several cases can be extrapolated to quite many other species of the genus *Tityus*. For this reason, but also because we are celebrating the centennial anniversary of *T. serrulatus*, I decided to resume here a number of scattered information previously published and also to suggest some neglected aspects of the possible evolution of this species.

## A summary of the historical aspects concerning *Tityus serrulatus*

*Tityus serrulatus* was described 100 years ago, and can now be considered as an ‘old’ species. Nevertheless, a paradox concerning the discovery of this species remains: Why had this common species not been described before 1922? After all, quite many species have been described, including several that belong to the genus *Tityus*, during the gold age of taxonomy, which took place in the last quarter of the 19^th^ century [19].

**Figure 2.**
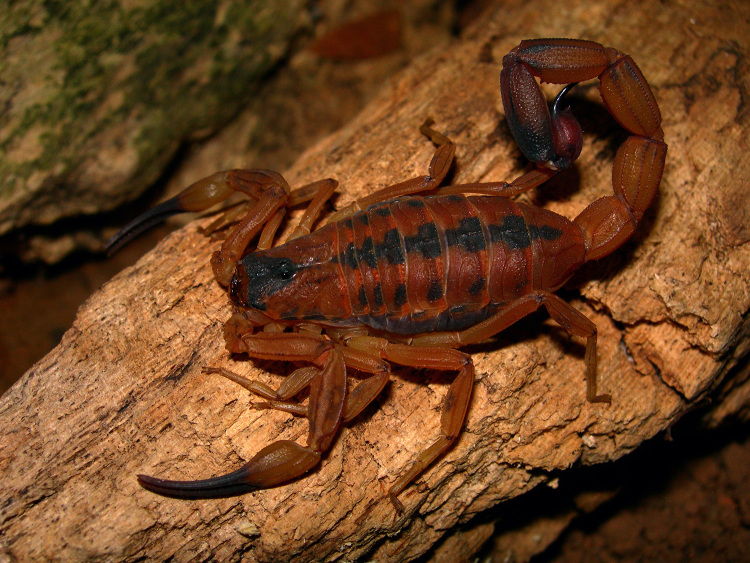
*Tityus stigmurus*. Female from Bahia state, Brazil (photo courtesy of Tiago J. Porto).

Several decades before the description of *T. serrulatus*, a similar species, *Tityus stigmurus* (Thorell, 1876) was described from the state of Pernambuco [20]; a species taxonomically associated with *T. serrulatus*. *T. stigmurus* (Fig. 2) was originally placed in another genus, *Isometrus* Ehrenberg, 1828 and later transferred to the genus *Tityus*. These forms of taxonomic indecisions were common in the 19^th^ century. Most certainly, the region of Pernambuco, as most coastal areas of Brazil, have been earlier prospected in comparison with the most interior zones of the country. Still, early expeditions such as that of Spix and Martius took place during the first half of the 19^th^ century and were largely accomplished in Central Brazil [13,21]. However, no specimen associated to *T. serrulatus* was ever reported.

One of the most preliminary and precise reports on incidents caused by *Tityus* species in Brazil was the thesis developed by Maurano [22]. In this monograph Maurano [22] refers mainly to *Tityus bahiensis* (Perty, 1833), an already common species in the state of São Paulo. Besides, he makes also reference to the presence of *T. stigmurus* in the state of Minas Gerais but his taxonomic skills were poor so this classification could be incorrect. Nevertheless, another author Vellard [23], in his studies about the scorpions of the state of Goiás, cited the presence of *T. stigmurus* in the city of Catalão. He also suggested that this population from the state of Goiás could represent one intermediate form between that of the Northeast of Brazil (*Tityus stigmurus* typicus), and the one from the Southeast (*Tityus serrulatus* typicus). Moreover, in his opinion, *Tityus serrulatus* should not be considered as a different species from *Tityus stigmurus*, but only as the most common form present in the southern range of the distribution of the species. I will return to this taxonomic aspect in a subsequent section.

**Figure 3.**
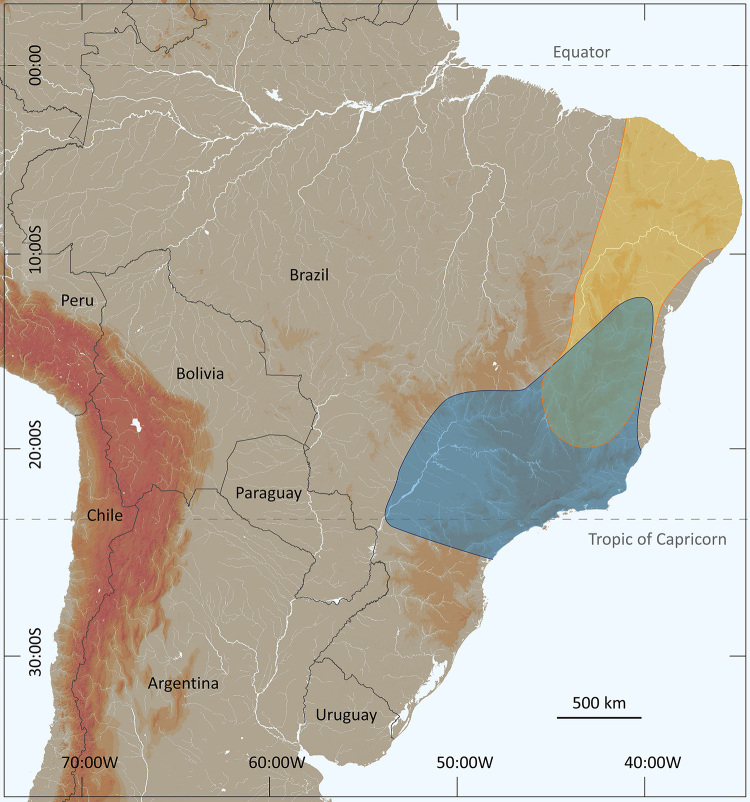
Map of eastern South America and Brazil showing the current distribution range of *Tityus stigmurus* (yellow) and *Tityus serrulatus* (blue), with a zone of sympatry (gray).

In a preliminary approach, Lourenço [24] suggested that *T. serrulatus* was closely related to *Tityus stigmurus* (Thorell) a bisexual species with a distribution further to the north compared to that of *T. serrulatus*. Several other authors however, have refused categorically to recognize the existence of a past southern distribution of *T. stigmurus* covering the present geographic range of *T. serrulatus* [25,26,27]. Others affirm that, before 1920, *Tityus stigmurus* was a common species in the central and southern regions of Brazil in the states of Minas Gerais, São Paulo and Goiás [2,23]. This question remained without a precise answer, and presently the two species show a sympatric zone only in the states of Minas Gerais (northern range) and Bahia (Fig. 3). Consequently, how to explain the geographical expansion of *Tityus serrulatus* in only one and half century?

## The possible model of geographic expansion of *Tityus serrulatus*

**Figure 4.**
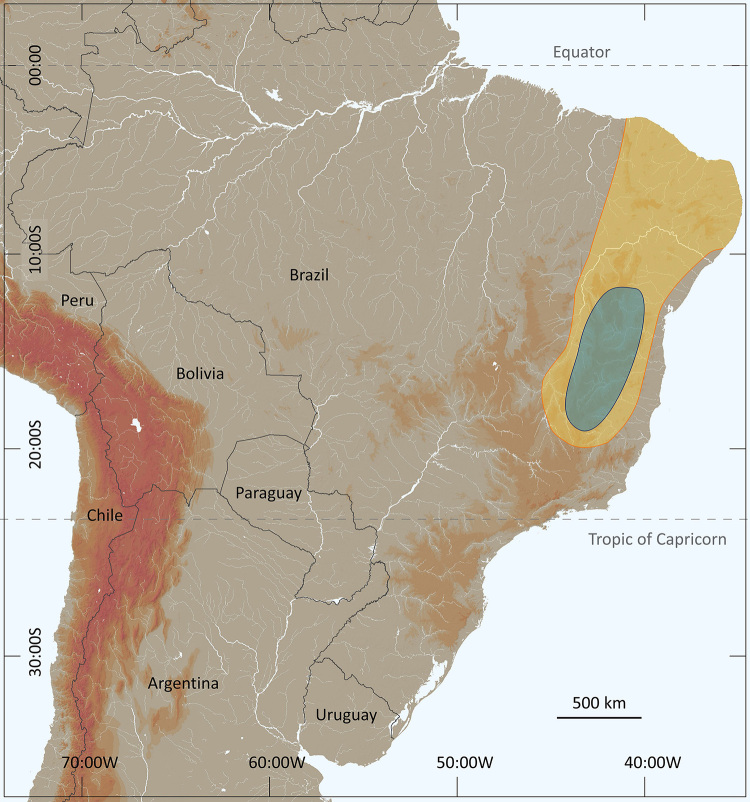
The suggested distributions of *Tityus stigmurus* (yellow) and *Tityus serrulatus* (blue) during the 16^th^ and 17^th^ centuries.

In the recent past, probably during the 16^th^ and 17^th^ centuries, the distributions of sexual populations of *Tityus serrulatus* and *Tityus stigmurus* most probably ranged from what is today the state of Minas Gerais to the northeastern region of Brazil (Fig. 4). The precise period and location when and where the parthenogenetic population of *Tityus serrulatus* “appeared” is particularly difficult to define. Nevertheless, it seems possible to suggest that this population was already present in the region of Minas Gerais even before the 18^th^ century; its presence however, was most certainly extremely inconspicuous (Fig. 4). 

At the beginning of the 18^th^ century, an important development was engendered by the Portuguese (especially in their search for gold) with the foundation of towns such as Curral d’El Rei and Vila Rica de Ouro Preto [28]. This entire natural region was previously composed of what could be defined as “metaclimax environments” and these natural habitats strongly suffered from human activities and turned to new modified environments, mostly presenting condition of disclimax. The formation of these modified environments favored the discrete parthenogenetic population of *Tityus serrulatus*, much more opportunistic than the sexual ones, to colonize the newly created modified habitats. 

**Figure 5.**
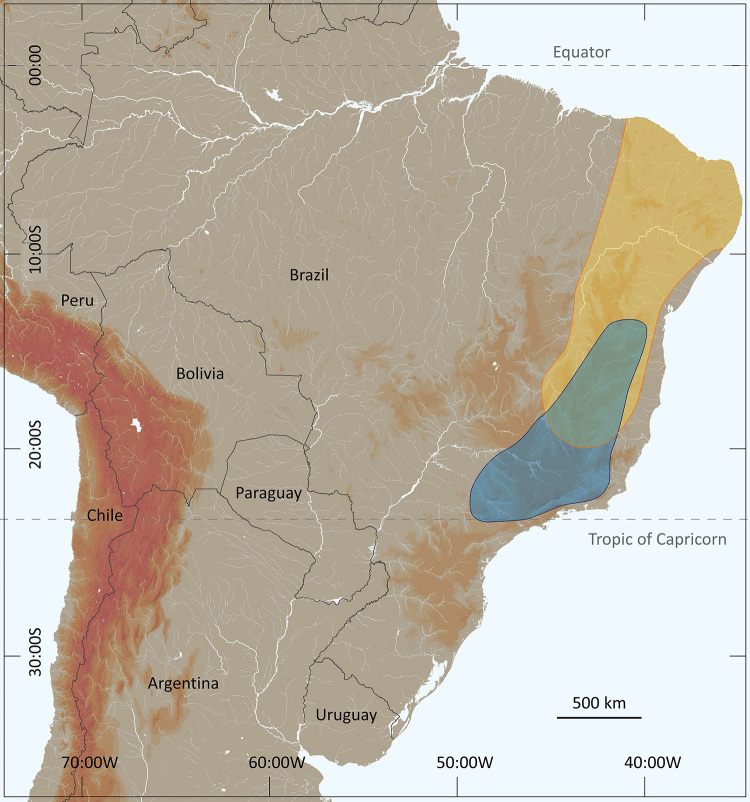
The suggested distributions of *Tityus stigmurus* (yellow) and *Tityus serrulatus* (blue), circa the 1920s, with a zone of sympatry (gray).

The expansion of human colonization toward the west and north resulted in a significant regression of the original bisexual populations of both *Tityus serrulatus* and *Tityus stigmurus,* which were gradually replaced by asexual populations of *Tityus serrulatus*. The asexual or parthenogenetic populations usually colonize urban areas (cities and towns), and can easily be transported by human activity from old to new cities. The progressive expansion of parthenogenetic populations of *T. serrulatus* was then observed in artificially created cities such as Belo Horizonte in Minas Gerais and Goiânia in Goiás during the first half of the 20^th^ century (Fig. 5). A more marked example comes from the city of Brasília, which was equally artificially constructed during the 1950s. In this precise case, the pace of the invasion by *T. serrulatus* was measured. During the 1970s, a first inventory study was done for the scorpion fauna of the Federal District region and no specimen of *T. serrulatus* was observed [29]. Subsequently, during the 1990s, a second inventory was performed and the presence of *T. serrulatus* was clearly confirmed in the urban areas of the city [30]. The comparative results demonstrated the possible invasion by asexual elements, probably from Minas Gerais and Goiás, in less than 20 years (Fig. 3).

## Ecological factors directly associated with the raising of asexual populations

**Figure 6.**
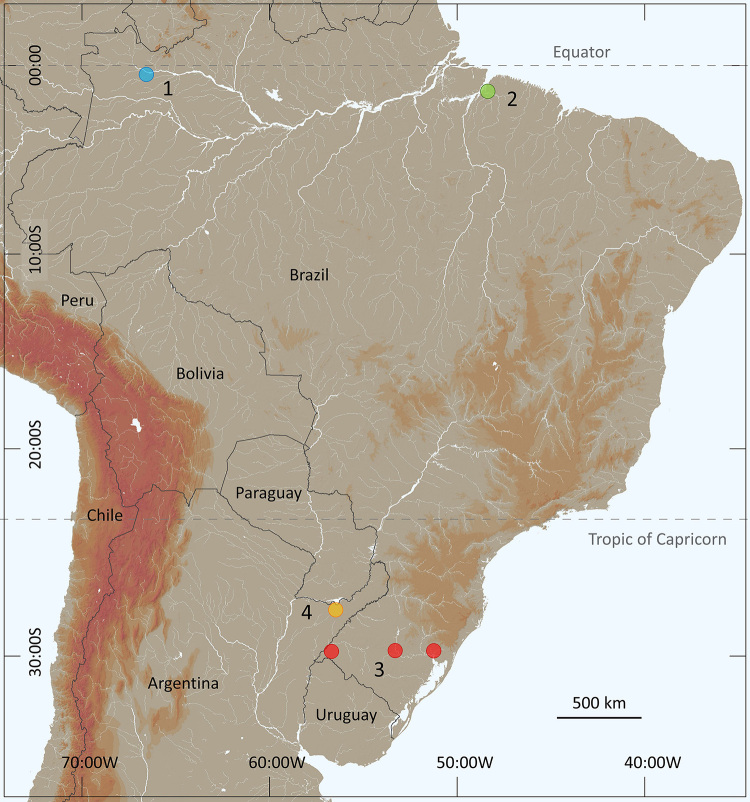
Sporadic records of *Tityus serrulatus* in locations out of the normal range of distribution of the species: **(1)** São Gabriel da Cachoeira (Amazonas state); **(2)** Belém (Pará state); **(3)** locations in the state of Rio Grande do Sul; and **(4)** Argentina.

Several authors have noted that parthenogenetic animals have a tendency to appear in environments that are different from those inhabited by their bisexual relatives [31-34]. Moreover, opportunistic (parthenogenetic) species have dispersion and colonizing potential much higher than that of equilibrium species. The asexual populations of *Tityus serrulatus* most certainly combine these two characteristics and clearly conform to the above predictions. The distribution of *T. serrulatus* in what are today the states of Minas Gerais and Bahia, was most certainly restricted some two or three centuries ago (Fig. 4), contrasting to its current wide distribution over a large region of the southeast and even south of Brazil (Fig. 3). The species is also reaching some bordering countries such as Bolivia and Argentina [10,35]. More recent records from some north regions of Brazil (Fig. 6) can however, be defined as sporadic [36-38]. 

The massive geographical expansion of asexual forms of *T. serrulatus* is undoubtedly related to human action, which introduced the scorpions into newly created cities and towns. Consequently, newly established human communities can be invaded in only a few years after their foundation, although the surrounding natural areas composed of savannas or forests are generally devoid of these invaders. The creation of new habitats suitable for colonization by *T. serrulatus* may be compared with the opening of clearings in dense primary forests [39]. The metaclimax savannas and forests in Brazil clearly represent these primary vegetation formations and new towns and cities are comparable with opened clearings. In both cases, the new environments are localized and occur in disclimax situations in which the process of secondary succession takes place. The new towns are, in many cases, separated by several hundred kilometers. Consequently, the country between them remains almost pristine and opportunistic species do not reach and colonize it.

The process of expansion of *Tityus serrulatus* usually occurs in steps. When scorpions are transported by anthropic agents, via roads or rail, and reach newly created human environments, they establish new colonies. These act as islands inside the metaclimax formation of savannas and forests. This process is greatly facilitated by the biological characteristics of *T. serrulatus*: (i) strong dispersal ability, (ii) a very high reproductive potential, (iii) a large reserve of populations, (iv) a great capacity to forage on prey resources that may be abundant, although not specific, and which are localized in a disclimax environment [39].

## The taxonomic positions of the elements belonging to the complex *Tityus stigmurus*

It is out of the scope of this limited note to bring major considerations on the taxonomy of the genus *Tityus*. Nevertheless, *Tityus serrulatus* does belong to a group or complex of species as already defined by Mello-Leitão [40] and Lourenço [24], and even previously suggested by Kraepelin [41]. The problem remains the precise definition of this group or complex and more than anything else, the precise definition of the relationships existing among the elements within the complex. After the redefinition of the complex by Lourenço [24] the problematic relationships among the elements of the group leaded to some empirical suggestions and the definition of varieties, which in fact don’t even have a nomenclatural status [42]. At present, it seems that this was not the good choice to clarify the possible relationships. Consequently, it seems reasonable to maintain the composition of the group, as defined by Lourenço [24], including some new described species and revalidating others placed in synonymies without full justifications [15]. 

**Figure 7.**
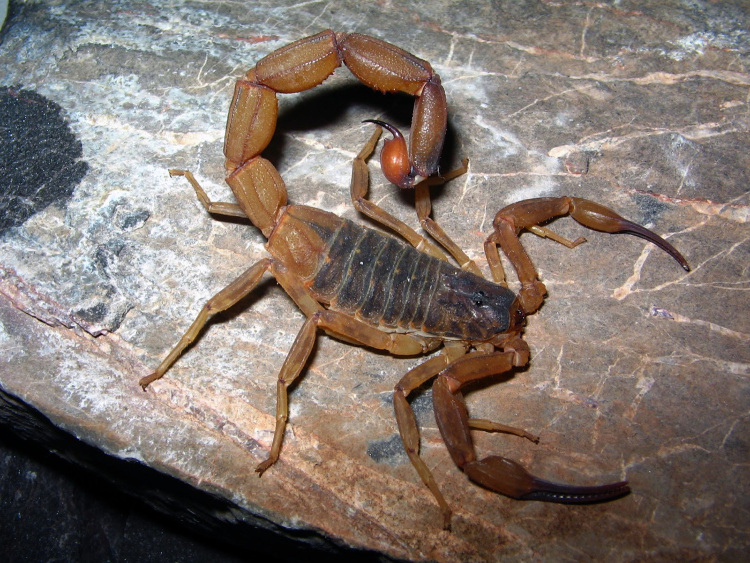
*Tityus lamottei*. Male from Bahia state, Brazil (photo courtesy of Victor Ghirotto).

**Figure 8.**
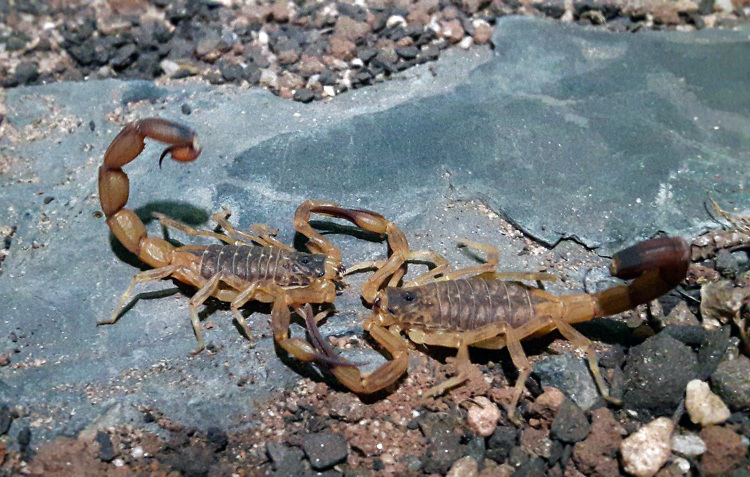
Mating behavior in *Tityus lamottei* (photo courtesy of Victor Ghirotto).

This decision concerns mainly two species. *Tityus lamottei* Lourenço, 1981 (Fig. 7), previously assumed as a possible synonym of *Tityus serrulatus* and *Tityus acutidens* Mello-Leitão, 1933, equally placed in the synonymy of *T. serrulatus* by Souza et al. [15]. For *T. lamottei*, we presently know (unpublished data) that its population is fully composed of sexual forms that cannot breed with *T. serrulatus* (Fig. 8). As for the second species, *T. acutidens* has been a source of confusion since its description. Supposedly collected in the Island of Bananal, its description was based on a poorly preserved specimen [24]. In addition, several months of field studies in the Island of Bananal, performed by myself, never revealed any specimen of this species. More recent studies suggested that the species was certainly collected in the state of Goiás [43]. Nevertheless, it sounds obvious that in the early 1930s *T. serrulatus* could not be present in the region of Goiás, therefore the association of both species sounds unfunded. For this reason, *T. acutidens* is revalidated at present.

**Figure 9.**
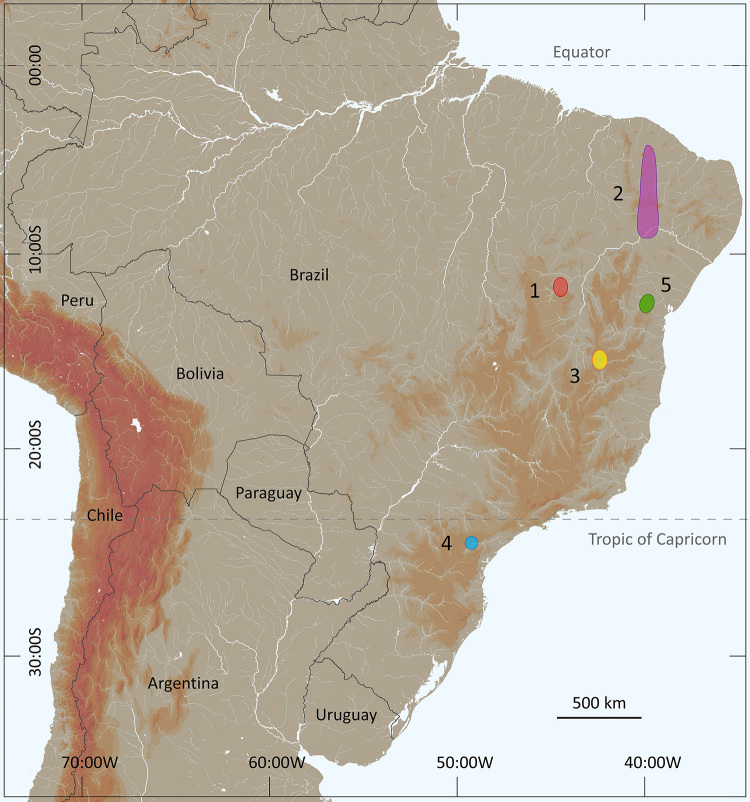
Known distributions of the species associated with the complex or group *Tityus stigmurus* presenting unequivocal original localities: **(1)** *Tityus lamottei*, **(2)** *Tityus martinpaechi*, **(3)** *Tityus melici*, **(4)** *Tityus pintodarochai*, **(5)** *Tityus aba*.

The present composition of a complex or group *Tityus stigmurus* can be defined as follows: *Tityus stigmurus* (Thorell, 1876); *Tityus serrulatus* Lutz & Mello, 1922; *Tityus acutidens* Mello-Leitão, 1933; *Tityus lamottei* Lourenço, 1981; *Tityus martinpaechi* Lourenço, 2001; *Tityus melici* Lourenço, 2003; *Tityus pintodarochai* Lourenço, 2005; and *Tityus aba* Candido, Lucas, Souza, Diaz & Lira da Silva, 2005 (Fig. 9). The inclusion of *Tityus kuryi* Lourenço, 1997 in this complex by Souza et al. [15], probably based on the single presence of serrulas in the metasomal segments, seems to be unjustified. Serrulas are equally present in other *Tityus* species [44]; therefore, this last species is for the moment excluded from the group.

## How is polymorphism defined within some *Tityus* populations?

Most populations in Brazil and other tropical South American countries consist of monomorphic species. One interesting example is provided by two related species from the Cerrados of Central Brazil. A statistical study of traits such as morphometric variation and patterns of pigmentation of *Tityus fasciolatus* Pessôa and *Tityus charreyroni* Vellard [19,45] showed no variability inside their respective populations. These two-sibling species are both inhabitants of savannas, but occupy different and quite specific microhabitats. *T. fasciolatus* is a termitophilous scorpion, whereas *T. charreyroni* lives under stones in a region west of that occupied by *T. fasciolatus*. The area and range of distribution of each population depends upon the existence and distribution of the specific microhabitat [19].

Examples of polymorphic species are, however, perfectly known. *Tityus bahiensis* (Perty) appears as a clinal polymorphic species with a very regular gradient. The observed pattern was first interpreted as polytypic [46,47], with subpopulations containing subspecies. *T. bahiensis* is very common in the south-eastern region of Brazil, and the global population shows a clinal gradient in their patterns of body pigmentation [19,46,47]. The study of specimens from many localities confirmed, however, that *T. bahiensis* is clearly a polymorphic species.

A second example concerns *Tityus costatus* (Karch), species distributed in the Atlantic forest of Brazil [13] and presenting a mosaic type polymorphism. The species ranges from the states of Espírito Santo and Minas Gerais to Rio Grande do Sul in the southern part of the Atlantic forest and shows a considerable variability in its pigmentation pattern. Two morphological models were defined: maculata and trifasciata with a gradient of intermediate forms. No correlations could be drawn between latitudinal gradient and the pattern of pigmentation [48], but further analysis between environmental factors and pigmentation patterns suggests that the maculata form occurred in sites ranging from sea level up to 1000 m, while the trifasciata form was found at sites above 1000 m. The relief of this part of the Atlantic forest is quite irregular and changes from sea level to more than 2000 m, which may occur over distances of less than 30 to 40 km. Consequently, the polymorphic pattern observed for *T. costatus* was defined as of the mosaic type.

Another example of polymorphism is the one presented by *Tityus silvestris* Pocock, species widely distributed in Amazon and French Guiana forests. In the case of this species, the variation concerns morphometric values that can be markedly distinct over the range of distribution. This variation showed little geographical correlation [49], and the same type of pattern was previously observed by botanists and termed “ochlospecies” [50]. According to Prance [51], “ochlospecies” are common in large genera containing over 100 species, which is the case of the genus *Tityus*. In Amazon and French Guiana, dry periods took place during palaeoclimatic episodes of the Pleistocene and forest cover was reduced to small patches and became fragmented with isolated allopatric populations of scorpions. Such isolated populations of ecologically adaptable species rapidly recolonized the forest that was re-established during the following wet episodes. Consequently, previously isolated populations became contiguous. In many cases, temporary reproductive isolation did not produce genetic incompatibility, such as for woody plants and scorpions. Only minor morphological differences evolved, and when species reunited, geographically variation was no longer well correlated. Prance [51] suggested that this type of variation is attributable to recent Pleistocene climate changes, and not to actual speciation.

## Perspectives: possible polymorphic variations within parthenogenetic populations

Naturally, all the cited examples of polymorphism are exclusively related to sexual populations. However, one question can be addressed: Can mutations inside isolated individuals of an asexual or parthenogenetic population equally conduct to polymorphic variations? This is a kin question involving directly the parthenogenetic *T. serrulatus*. The isolated individuals of an asexual population normally reproduce as clones but other more complex mechanisms may also take place inside the populations such as automictic processes. Consequently, some populations may be asexual, but not clonal [52-54]. In the case of clonal reproduction, even if the mutations cannot spread via sexual recombination, the clones produced by a female *T. serrulatus* will equally carry the mutations that will be present in the following generations. These mutations can, in many cases, positively select individuals of a given population to better adapt to new and distinct habitats.

**Figure 10.**
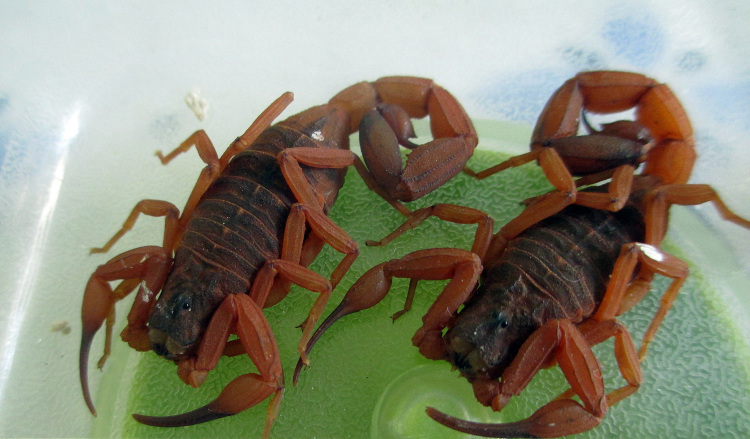
Specimens of *Tityus serrulatus* collected in the city of Americana in the state of São Paulo, Brazil, presenting a “reddish” pattern of pigmentation (photo courtesy of José Brites-Neto).

Some empirical observations, reported for the populations of *T. serrulatus* present in the city of Americana, in the state of São Paulo, suggested the presence of two patterns of coloration (Dr. José Brites-Neto, personal communication). One globally yellowish, typical of the original populations and the second strongly “reddish” (Fig. 10). However, no further studies are yet available concerning this phenomenon.

It is also important to recall that the original population of *T. serrulatus* inhabit and forage in hot and dry tropical savannas [55]. The artificial transportation of elements of these tropical populations, via human agency, to more temperate zones such as the South of Brazil or Argentina, necessarily requires a process of re-adaptation to these colder and wet habitats. In particular, the reproductive strategies of the species may have to readapt. In its native habitat, *T. serrulatus* can produce more than one brood per year, with an average embryonic period of 2.5 to 3 months, and post-embryonic development periods of only 1 to 1.5 years [56,57]. In more cold conditions, reproduction may be limited to the warmer periods (spring and summer) and possible diapause processes may take place during winter [56-58].

New genetic studies are yet necessary to test the suggested hypothesis. Nevertheless, this new proposed model has already some consequences on the way how *Tityus serrulatus* should be globally regarded and mostly its implications on the biochemical and immunological research conducted with this species. The observed variation in the pigmentation patterns could also suggest the existence of biochemical polymorphisms of venom toxins. This has a major importance in the study of these venoms, which should be geographically defined with precision.
